# Zuogui Wan ameliorates high glucose-induced podocyte apoptosis and improves diabetic nephropathy in db/db mice

**DOI:** 10.3389/fphar.2022.991976

**Published:** 2022-11-01

**Authors:** Bingbing Zhu, Ji Fang, Zhengcai Ju, Ying Chen, Li Wang, Hao Wang, Lina Xing, Aili Cao

**Affiliations:** ^1^ Department of Nephrology, Shanghai Sixth People’s Hospital Affiliated to Shanghai Jiao Tong University School of Medicine, Shanghai, China; ^2^ Department of Nephrology, Putuo Hospital, Shanghai University of Traditional Chinese Medicine, Shanghai, China; ^3^ Shanghai Key Laboratory of Complex Prescriptions and MOE Key Laboratory for Standardization of Chinese Medicines, Institute of Chinese Materia Medica, Shanghai University of Traditional Chinese Medicine, Shanghai, China

**Keywords:** Zuogui Wan, diabetic nephropathy, podocytes, apoptosis, reactive oxygen species

## Abstract

Zuogui Wan (ZGW), a well-known traditional Chinese medicine (TCM), has been used to nourish “Kidney-Yin” for a long time in China, implying a protective effect on the kidney. The aim of the present study is to investigate the effect of ZGW on high glucose-induced podocyte apoptosis and diabetic nephropathy (DN) in db/db mice. ZGW (1 g/kg^−1^/day^−1^) was administered intragastrically to db/db mice for 8 weeks. HPLC was used for identifying the components of ZGW, biochemical and histopathological approaches were used for evaluating its therapeutic effects, and cultured mouse podocytes were used for further exploring its underlying mechanism *in vitro.* ZGW improved renal function and podocyte loss and also normalized kidney reactive oxygen species production in *db/db* mice. The cytotoxicity of ZGW on mouse podocytes was assessed by the LDH assay. The effect of ZGW on podocyte viability and apoptosis was determined with CCK-8 and Annexin-V/PI staining by treatment with high glucose. ZGW attenuated podocyte apoptosis, and oxidative stress was detected by the peroxide-sensitive fluorescent probe 2′,7′-dichlorodihydrofluorescein diacetate (DCF-DA) staining in a dose-dependent manner. Furthermore, ZGW decreased the expression of caspase-3 and phospho-p38 in both the kidney cortex and high glucose-treated podocytes. Thus, our data from *in vivo* and *in vitro* studies demonstrated that ZGW improved renal injury in diabetes by inhibiting oxidative stress and podocyte apoptosis.

## Introduction

Diabetic nephropathy (DN), one of the most common diabetic complications in diabetes, has become the most frequent cause of chronic kidney disease that leads to end-stage renal disease (ESRD) (Collaboration, 2020). Its pathogenesis can be attributable to various factors. Podocyte, a component of the glomerular basement membrane (GBM), plays a major role in maintaining the glomerular filtration barrier. Podocyte injury and loss result in glomerular disease and progression of renal failure (Naylor et al., 2021). In previous studies, it has been confirmed that high glucose-induced apoptosis and ROS production contribute to podocyte loss (Casalena et al., 2020; Li et al., 2021). Literature concerning Chinese medicine therapies for treating DN in recent years reveals that Chinese medicine has made some progress in the pathogenesis and treatment of DN based on syndrome differentiation (Yang et al., 2020; He et al., 2022).

As a traditional Chinese prescription described in the TCM monograph *Jing-Yue Quan Shu* by the distinguished physician Zhang Jing-Yue in the Ming Dynasty, ZGW has been long utilized clinically in the treatment of kidney Yin deficiency syndromes. It consists of eight Chinese crude drugs, namely, Rehmanniae Radix Praeparata, Dioscoreae Rhizoma, Lycii Fructus, Corni Fructus, Achyranthis Bidentatae Radix, Cervi Cornus Colla, Testudinis Carapacis et Plastri Colla, and Cuscutae Semen. It has been reported that ZGW can prevent glucocorticoid-induced osteoporosis in rats by upregulating the expression of the Wnt signal transduction pathway (Liu et al., 2011). It also relieves axonal injury and promotes axonal regeneration in rats with experimental autoimmune encephalomyelitis (Wang et al., 2010). However, the underlying activity of ZGW in preventing renal injury in DN has not been fully understood. In this study, we investigated the protective effects of ZGW on podocytes in mice with DN and explored the molecular and cellular mechanisms underlying these effects.

## Materials and methods

### Chemicals and reagents

Eight individual medicinal materials of ZGW, including Rehmanniae Radix Praeparata, Dioscoreae Rhizoma, Lycii Fructus, Corni Fructus, Achyranthis Bidentatae Radix, Cervi Cornus Colla, Testudinis Carapacis et Plastri Colla, and Cuscutae Semen (See also in Table 1) were obtained from Leiyunshang Pharmaceutical (Shanghai, China) and identified by experts in pharmacognosy from the Institute of Chinese Materia Medica, Shanghai University of Traditional Chinese Medicine. The herbs were subjected to reflux extraction with water for 1.5 h twice to obtain the water decoction and concentrate. Then, 95% ethanol was added to regulate the concentration of ethanol to be at 80%. The supernatant was dried and mixed with Colla Cornus Cervi and Colla Plastri Testudinis. The resulting dry extract was stored at −20°C until usage. A total amount of 1.0 g extract powder equals to 3.0 g raw herbs.

**TABLE 1:** Zuogui Wan’s composition.

Chinese name	English name	Botanical name	Family	Weight (g)	Part used
Shu Di Huang	Rehmanniae Radix Praeparata	Rehmannia glutinosa Libosch	Scrophulariaceae	200	Root
Shan Yao	Dioscoreae Rhizoma	Dioscorea opposite Thunb.	Dioscoreaceae	100	Rhizome
Gou Qi Zi	Lycii Fructus	Lycium barbarum L.	Solanaceae	100	Fruit
Shan Zhu Yu	Corni Fructus	Cornus officinalis Sieb. et Zucc.	Cornaceae	100	Sarcocarp
Niu Xi	Achyranthis Bidentatae Radix	Achyranthes bidentata Bl.	Amaranthaceae	75	Root
Lu Jiao	Cervi Cornus Colla	Cervus elaphus Linnaeus	Cervidae	100	Horn
Gui Ban Jiao	Testudinis Carapacis et Plastri Colla	Chinemys reevesii (Gray)	Emydidae	100	Shell
Tu Si Zi	Cuscutae Semem	Cuscuta chinnensis Lam.	Solanaceae	100	Seed

CCK-8 was purchased from DojindoLaboratorise (JPN), and the Annexin V/PI Apoptosis Detection kit from Biovision (Switzerland). LDH, SOD and CAT kits were obtained from Cayman Chemical (United States). DCF-DA was from Invitrogen (United States). Fasting blood glucose levels were measured with the Omron HEA-230 Glucometer using one drop of tail blood (JPN). Urinary albumin and creatinine were monitored with the ELISA Kit (Biovision, United States), and the urinary albumin to creatinine ratio (uACR) was used to observe the kidney function. The primary antibodies phospho-ERK, ERK, phospho-JNK, JNK, phospho-p38, p38, caspse-3, and ß-actin for western blot analysis were purchased from Cell Signaling Technology (United States). The primary antibody, WT-1, was obtained from Abcam (United States). Alexa Fluor 488-labeled goat anti-rabbit IgG secondary antibody for immunofluorescence staining and goat anti-rabbit HRP secondary antibody for western blot were obtained from Jackson ImmunoResearch (United States). The RIPA lysis buffer and BCA Assay kit were obtained from Beyotime (China).

### HPLC analysis

**FIGURE 1:**
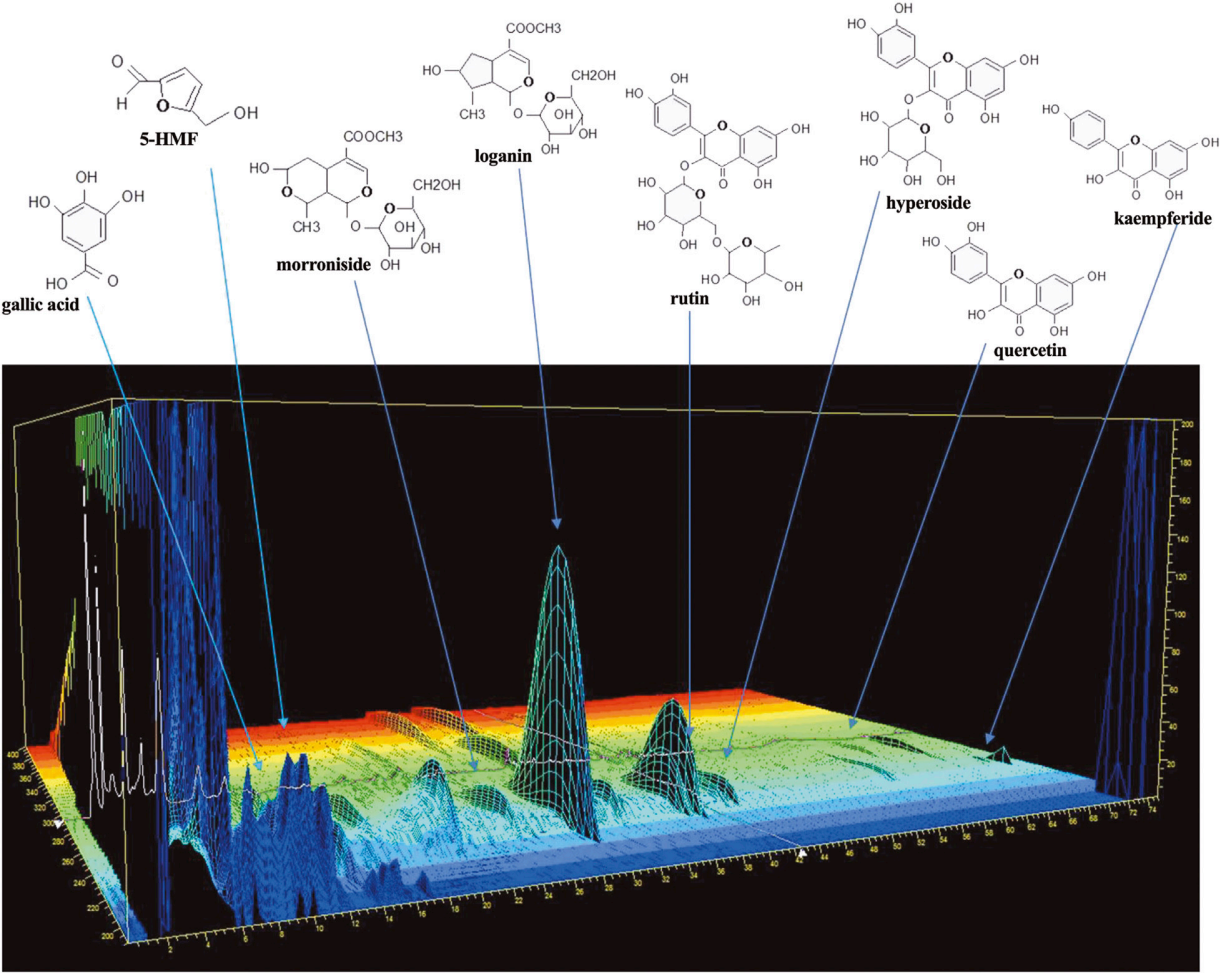
Three-dimensional HPLC profile of Zuogui pill.

A three-dimensional high-performance liquid chromatography (3D-HPLC) profile of ZGW is shown in Figure 1. The analysis procedure was conducted according to the previous study (Liang et al., 2009).

### Animal groups and treatments

Six-week-old male nondiabetic db/m and diabetic db/db mice (C57BLKS/J-leprdb/leprdb) were obtained from the Model Animal Research Center of Nanjing University. The experimental protocol was approved by the ethics committee of Putuo Hospital, Shanghai University of Traditional Chinese Medicine. The mice were given free access to food and water and maintained in conditions of a 12/12 h light/dark cycle and controlled temperature (23°C ± 2), and humidity (55 ± 5%). At 8 weeks of age, they were divided into three groups: Group 1, nondiabetic control db/m mice (*n* = 10); Group 2, diabetic nephropathy db/db mice (*n* = 10); and Group 3, db/db mice treated *via* intragastrical administration with 1 g/kg/day of ZGW dissolved in 0.5% carboxymethyl cellulose (CMC). Group 1 and 2 received 0.5% CMC for 8 weeks. The dose we used was calculated from the equivalent conversion by the body surface area between animals and humans according to the recommended daily human dosage. Mice were housed in individual metabolic cages for a 24 h urine collection. Body weight, water intake, blood glucose, food intake, and ACR were detected at 4-week intervals. At the end of the study, the animals were killed, and both kidneys were removed immediately. The renal cortex was divided into five parts for histological examination, immunohistochemistry, electron microscopy, ELISA, and western blot.

### Histopathological, immunofluorescence, and electron microcopy evaluation

For histological analysis, the renal cortex was fixed overnight in 4% PFA and embedded in paraffin. Subsequently, paraffin-embedded samples were cut into 4-μm thick slices and stained with periodic acid-Schiff (PAS). For immunofluorescence, kidney samples were transferred to 18% sucrose overnight and then flash-frozen in OCT medium. For electron microscopy, kidney samples were fixed in 2.5% glutaraldehyde.

### Cell culture and Zuogui Wan treatment

Conditionally immortalized mouse podocytes were kindly provided by Dr. Peter Mundel and were conducted as previously described (Saleem et al., 2002). All cells were grown in a dish coated with type l collagen (Invitrogen, United States). Podocytes were incubated at 33°C with 10 U/ml mouse recombinant interferon-γ (IFN-γ, Peprotech, United States), 10% heat-inactivated fetal calf serum, 100°U/ml penicillin and 100 μg/ml streptomycin in RMPI 1640 for proliferation. At confluence podocytes were incubated at 37°C for 14°days deprived of IFN-γ. Differentiated podocytes were cultured for 24 h in RMPI 1640 with 1% FCS before various experimental conditions. The cells were divided into normal glucose group, mannitol group incubated in 5 mM d-glucose with 25 mM D-mannitol (Sigma, United States), high glucose (HG) group incubated in RPMI 1640 containing 30 mM d-glucose (Sigma, United States), and ZGW group incubated in HG medium and treated with different concentrations of ZGW. All of the experimental groups were cultured in triplicate.

### Determination of reactive oxygen species and oxidative stress

ROS generation was measured as previously described (Huntosova et al., 2021). Briefly, podocytes were incubated for 45 min at 37°C with 50 μM 2′,7′-dichlorodihydrofluorescin. The DCF fluorescence was determined in a multiwall fluorescence plate reader (Thermo Scientific Varioskan Flash, United States). The oxidative stress markers, including activities of catalase (CAT) and superoxide dismutase (SOD) in kidney cortex and cultured mouse podocytes, were measured using the assay kits according to the manufacturer’s instructions.

### Lactate dehydrogenase assay

Necrotic cell death was assessed by the release of lactate dehydrogenase (LDH) from the cytosol of the damaged cells into the supernatant. The LDH cytotoxicity detection kit was performed according to the manufacturer’s instructions.

### Cell viability assay

Cell viability was assessed by the CCK-8 kit according to the manufacturer’s protocol. Podocytes were plated in 96-well plates at 2 × 10^4^ cells per well and differentiated at 37°C for 12 days. Then, cells were treated with or without Zuoggui Wan for 12–48 h. CCK-8 was added to each well and incubated for 2 h. The cell viability was determined by absorbance at a wavelength of 450 nm. The relative cell viability was calculated from: Cell Viability (%) = (1 – mean absorbance of cells in sample groups/mean absorbance of cells in control groups) ×100.

### Apoptosis assay

Apoptosis was determined by Annexin V/propidium iodide (PI) as described in the manufacturer’s protocol. After 24 h of treatment, podocytes were washed twice with cold phosphate-buffered saline (PBS) on ice, trypsinized, and pelleted by centrifugation at 1000 rpm for 5 min. Then, they were resuspended in binding buffer and stained with annexin V/PI and analyzed for apoptosis using a FACS scan (Merk Millipore, United States).

### Western blotting

Kidney cortex or cultured podocytes were lysed in lysis buffer using a sonicator and then centrifuged at 12,000 g for 5 min at 4°C. The total protein concentration from the supernatant was determined by the BCA protein assay. Cell lysates (40 μg protein/lane) were resolved by electrophoresis on 10%–15% sodium dodecyl sulfate polyacrylamide gel electrophoresis (SDS-PAGE) and then transferred onto equilibrated polyvinylidene fluoride (PVDF) membranes. After blocking using 5% skimmed milk, the membrane was incubated with primary antibodies individually (1:1000 dilution for each) at 4°C overnight. The bound antibodies were incubated with horse-radish peroxidase-labelled goat anti-rabbit IgG and detected by the ECL plus Kit.

### Statistical analysis

All experiments were repeated at least three times. The mean ± standard deviation (SD) was determined for each group. Statistical analysis was performed with one-way analysis of variance (one-way ANOVA) and the Newman–Keuls multiple comparison test between three groups or an unpaired t test between two groups. Differences at *p* < 0.05 were considered statistically significant. Statistical analyses were conducted with Graphpad Prism software 5 software (United States).

## Results

### HPLC fingerprint of Zuogui Wan

The typical HPLC fingerprint of ZGW is shown in Figure 1. An effective and reliable method (Liang et al., 2009) has been established to analyze and quantify the constituents of ZGW. Ten compounds including gallic acid (203 μg/g), 5-HMF (1329 μg/g), Morroniside (632 μg/g), Sweroside (53 μg/g), Loganin (472 μg/g), Ecdysterone (15 μg/g), Rutin (17 μg/g), Hyperoside (68 μg/g), Quercetin (8 μg/g), and kaempferide (55 μg/g) were analyzed and quantified. In our study, by comparison with the retention times of the reference standards, eight compounds including gallic acid, 5-HMF, morroniside, loganin, rutin, hyperoside, quercetin, and kaempferide in ZGW were identified.

### Effect of Zuogui Wan on biochemical and physical parameters

**FIGURE 2:**
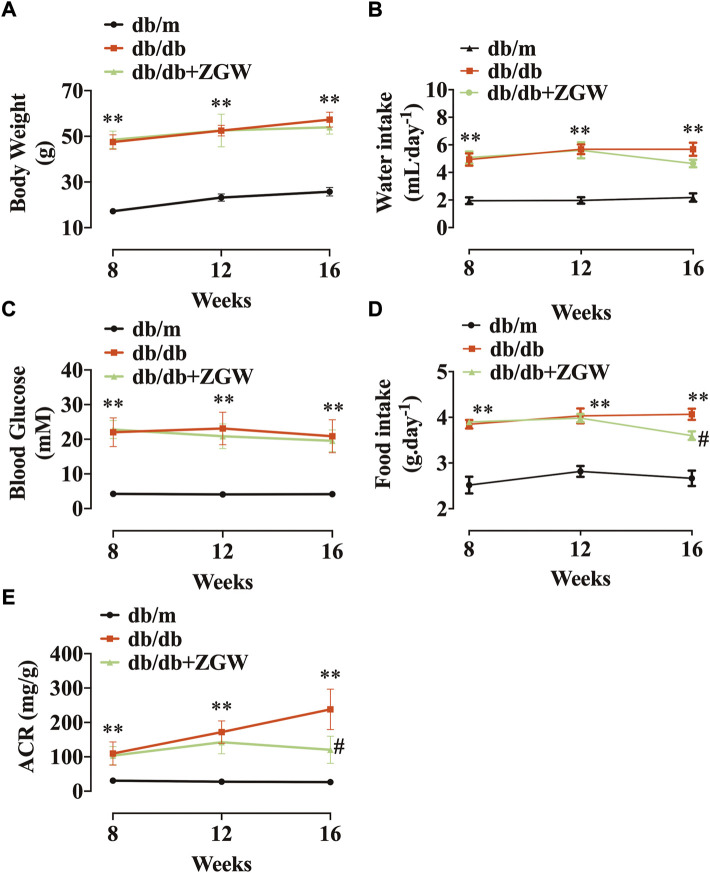
Effects of ZGW (ZGW) on metabolic parameters in db/db mice. Eight-week-old db/db mice were treated with ZGW for 8 consecutive weeks. **(A)** Body weight, **(B)** water intake, **(C)** blood glucose, **(D)** food intake, and **(E)** ACR. The results are mean ± SD. Statistical analysis was performed with one-way ANOVA and the Newman–Keuls multiple comparison test. ^**^ *p* < 0.01, compared with db/m mice. ^#^ *p* < 0.05 compared with db/db mice.

Db/db mice are used to model phases 1 to 3 of diabetes type II and obesity (King, 2012). During the experiment, diabetic db/db mice had higher body weight, blood glucose, food intake, and water intake compared with normal db/m mice. In db/db mice, treatment with ZGW failed to reduce body weight, water intake, and blood glucose (Figures 2A–C) but decreased food intake (Figure 2D). As compared to db/m mice, db/db mice developed time-dependent progressive ACR. Administration of ZGW significantly improved the development of albuminuria (Figure 2E).

### Zuogui Wan ameliorated renal histopathology in db/db mice

**FIGURE 3:**
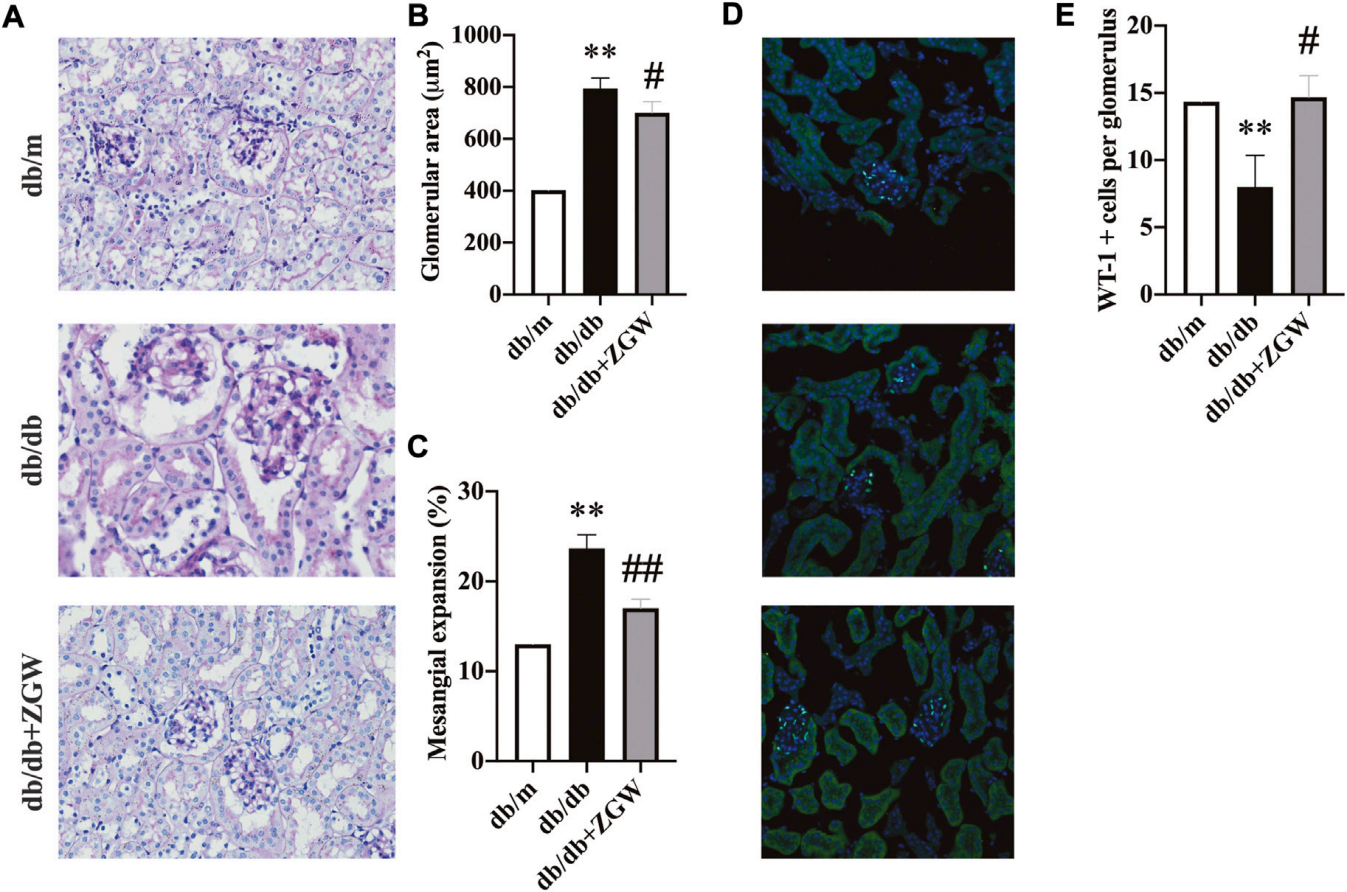
ZGW ameliorated renal histopathology in diabetic db/db mice. **(A)** Pathological changes of the kidney in different groups observed by PAS staining. **(B)** Glomerular size. **(C)** Mesangial expansion. **(D)** Immunofluorescence analysis of Wilms’ tumor suppressor gene 1 (WT-1). **(E)** Quantification of WT-1-positive podocyte number per glomerulus. Magnification: x200 in A and D. The results are expressed as the mean ± SD. Statistical analysis was performed with one-way ANOVA and the Newman–Keuls multiple comparison test. ^**^ *p* < 0.01, compared with db/m mice. ^#^ *p* < 0.05, ^##^ *p* < 0.01 compared with db/db mice.

Consistent with the attenuation of albuminuria, ZGW significantly ameliorated renal injury in db/db mice. Figure 3A shows representative renal pathology. Relative to nondiabetic db/m mice, mesangial expansion was severe in diabetic db/db mice. However, in the ZGW treatment group, mesangial expansion and glomerular hypertrophy were markedly improved. Semiquantitative data further confirmed these observations (Figure 3B,C).

Podocytes play an important role in glomerular function. Together with endothelial cells and mesangial cells, they form a glomerular filtration barrier, a key player in the development of proteinuria. Increasing evidence suggests that primary lesions in renal podocytes are the early cause of end-stage renal failure (Jefferson et al., 2011; Fogo, 2015). Wilm’s tumor 1 (WT-1) gene is expressed in podocytes throughout life and is used as a podocyte marker to evaluate the podocyte lesion (Guo et al., 2002; Su et al., 2010). In our experiment, immunofluorescence staining of WT-1 revealed marked podocyte lesion in db/db mice, while ZGW effectively prevented podocyte loss (Figure 3D,E).

### Zuogui Wan improved oxidative stress and the p38/MAPK signaling pathway in db/db mice

**FIGURE 4:**
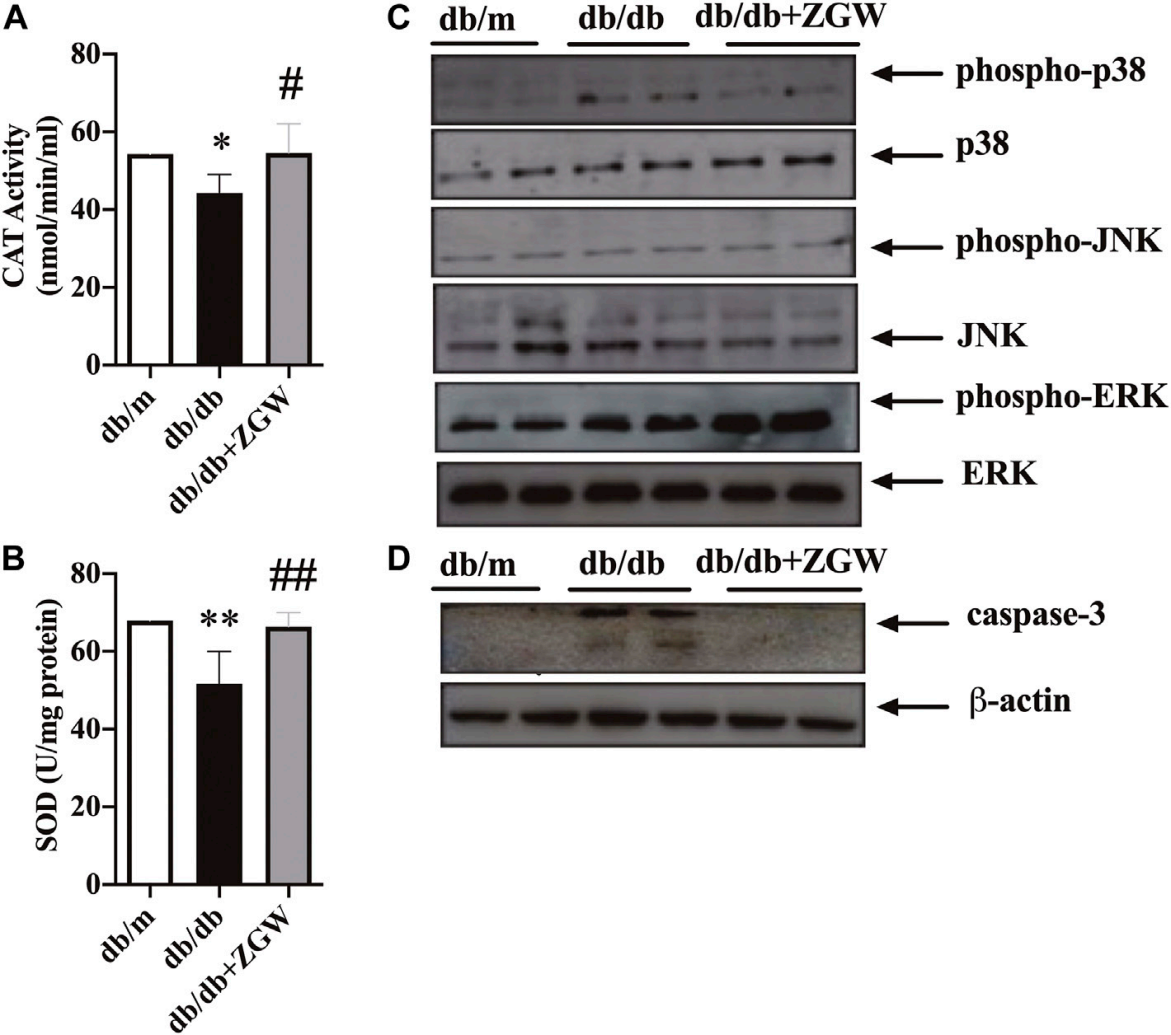
ZGW elevated activities of CAT and SOD, inhibited caspase-3, and regulated p38/MAPK signaling in db/db mice. The effects of ZGW on **(A)** CAT and **(B)** SOD were detected by ELISA. Representative immunoblots of MAPK signaling **(C)** and cleaved caspase-3 **(D)**. Statistical analysis was performed with one-way ANOVA and an unpaired t test. ^**^ *p* < 0.05, ^**^ *p* < 0.01, compared with db/m mice. ^#^ *p* < 0.05, ^##^ *p* < 0.01 compared with db/db mice.

Diabetes is a chronic disease; sustained hyperglycemia stimulates microvessels and macrovessels throughout the body (Barrett et al., 2017). Diabetes imbalances the production of reactive oxygen species (ROS) and thus results in increased oxidative stress. Chronic oxidative stress leads to cellular homeostasis and generates other ROS, creating further damage in DN (Han et al., 2018; Galvan et al., 2019). The antioxidant defense system of the cell is crucial for oxidative stress. In diabetes, the activities of antioxidant defense enzymes such as SOD and CAT are responsible for scavenging free radicals and regulating redox homeostasis (Linke et al., 2005). Hence, we examined whether improvement in renal function was ascribed to suppression of oxidative stress in the kidney cortex. As shown in Figure 4A,B, relative to nondiabetic db/m mice, CAT and SOD levels in diabetic db/db mice were markedly low but showed a significant increase by treatment with ZGW.

Oxidative stress activates the MAPK signaling pathway (Hole et al., 2013; Lu et al., 2021). ZGW decreased the activation of p38/MAPK rather than JNK and ERK (Figure 4C). The activation of MAPK in response to oxidative stress through the generation of ROS can have proapoptotic effects. Here, cleaved caspase-3 in db/db mice was increased and ZGW abolished this effect (Figure 4D), strongly suggesting the regulating role of ZGW in oxidative stress and oxidative stress-induced apoptosis.

### Zuogui Wan inhibited high glucose-induced apoptosis in podocytes

**FIGURE 5:**
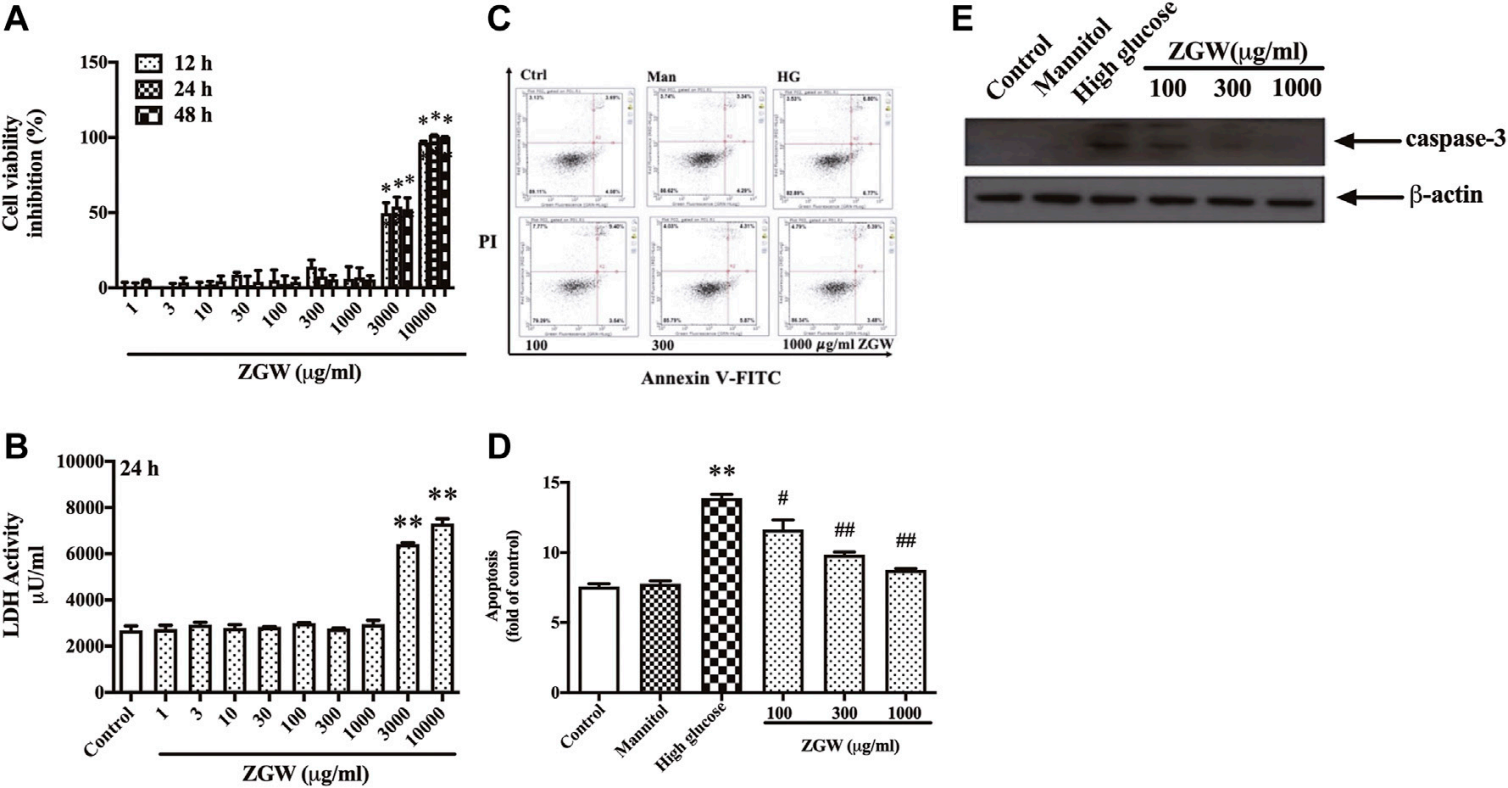
Effects of ZGW on viability, necrosis, and apoptosis. Podocytes were incubated with increasing concentrations of Zuogui pill (0, 1, 3, 10, 30, 100, 300, 1,000, 3,000, and 10,000 μg/ml). **(A)** Cell viability was examined using CCK-8 assay after 12, 24, and 48 h treatment with Zuogui pill. **(B)** Necrotic cell death was assessed by the release of LDH after 24 h treatment with Zuogui pill. **(C)** Podocytes were preincubated with Zuogui pill by 100, 300, and 1,000 μg/ml for 1 h then exposed to high glucose (30 mM). Effects of Zuogui pill on apoptosis were assessed by FACS after annexin V/PI staining. Representative flow cytometry results for podocytes under different culture conditions. **(D)** Semiquantitative data showing percentage of apoptotic podocytes. **(E)** Representative immunoblots of cleaved caspase-3. Statistical analysis was performed with one-way ANOVA and the Newman–Keuls multiple comparison test. ^**^ *p* < 0.01 compared with the control group. ^#^ *p* < 0.05, ^##^ *p* < 0.05 compared with the high glucose group.

In order to examine the effect of ZGW on cell viability, podocytes were treated with increasing concentrations of ZGW (0–10,000 μg/ml) for 12–48 h. As shown in Figure 5A, there were few dead podocytes at the concentration of 1,000 μg/ml, while 3,000 μg/ml ZGW significantly decreased the survival of podocytes. On the other hand, to evaluate ZGW-induced necrosis, the release of LDH in the extracellular medium was determined. As shown in Figure 5B, the level of released LDH was relatively low at a concentration of 1,000 μg/ml. Thus, 1,000 μg/ml was used as the highest concentration of ZGW for further study.

High glucose is a proapoptotic factor and leads to podocyte apoptosis by inducing oxidative stress (Wang et al., 2020). We further confirmed the protective effect of ZGW on high glucose-induced apoptosis in podocytes by FACS after Annexin-V-FITC and PI staining. As shown in Figure 5C,D, exposure to HG for 24 h resulted in significant apoptotic cell death relative to control cells. Mannitol had no effect on cell apoptosis. However, ZGW reduced the podocyte apoptosis at concentrations of 100, 300, and 1,000 μg/ml. Meanwhile, the activation of caspase-3 was inhibited by ZGW treatment in HG-triggered podocytes (Figure 5E).

### Zuogui Wan reduced HG-induced reactive oxygen species production, oxidative stress, and p38/MAPK signaling pathway in podocytes

**FIGURE 6:**
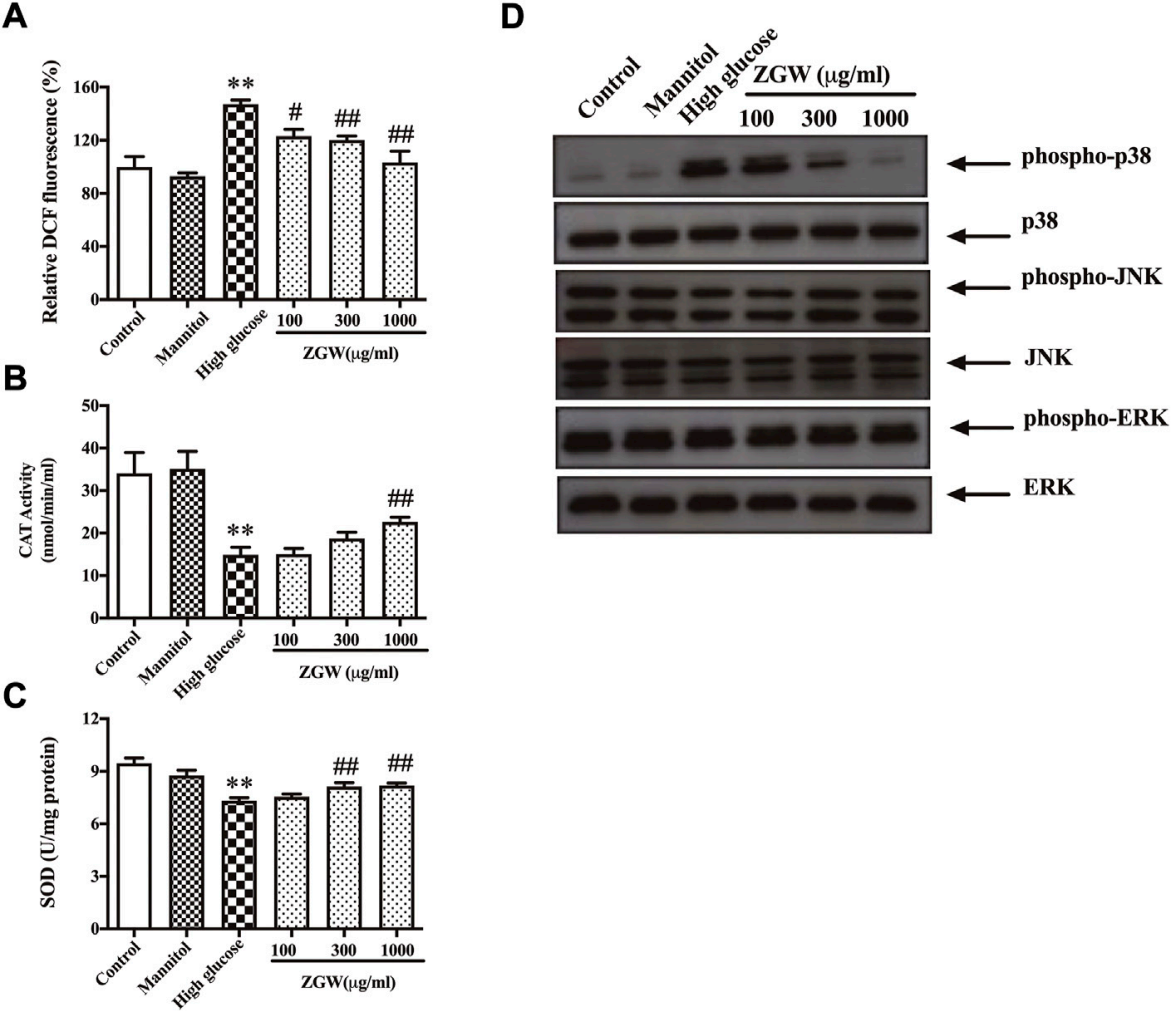
ZGW dose-dependently inhibited high glucose-induced ROS generation, elevated CAT, SOD, and regulated p38 MAPK in podocytes. Podocytes were preincubated with 100, 300, and 1,000 μg/ml ZGW for 1 h and then exposed to normal, mannitol (5 mM glucose and 25 mM mannitol), and high glucose (30 mM). The effects of Zuogui pill on ROS were determined by **(A)** DCF-DA staining. The effect of Zuogui pill on **(B)** CAT and **(C)** SOD was detected by ELISA. **(D)** Representative immunoblots of MAPK signaling. Statistical analysis was performed with one-way ANOVA and the Newman–Keuls multiple comparison test. ^**^ *p* < 0.01, compared with the control group. ^#^ *p* < 0.05, ^##^ *p* < 0.01 compared with high glucose.

Next, to evaluate the effects of ZGW on ROS generation, podocytes were stained with DCF-DA. Compared with the control group, exposure to HG for 24 h significantly induced ROS production. Mannitol kept ROS levels unchanged, while ZGW reduced HG-induced ROS production (Figure 6A). Moreover, the activities of SOD and CAT in cultured podocytes were measured as shown in Figure 6B,C. Exposure to HG for 24 h led to a remarkable decrease in both SOD and CAT levels, and preincubation with ZGW increased their levels. Meanwhile, HG resulted in elevated expression of phospho-p38 in podocytes, which was abrogated by ZGW treatment in a dose-dependent manner (Figure 6D).

## Discussion

Approximately one-third of patients with type 1 diabetes and half of patients with type 2 diabetes will develop DN. DN is the leading cause of CKD worldwide (Collaboration, 2020). Over time, high blood glucose and pressure can damage various areas of the body, including the cardiovascular system, and be the major cause of long-term kidney disease and ESRD. ESRD is the last stage of kidney disease and can lead to kidney failure and death (Thomas et al., 2015). The inability of kidney cells to downregulate glucose transport in response to high glucose levels leads to an overwhelming flux of intracellular glucose, which triggers the generation of pathogenetic mediators that contribute to the development of DN (Hong et al., 2018; Cao et al., 2021). Many steps can be taken to prevent or delay kidney damage. Due to reduced renal excretion, many antidiabetic drugs are either contraindicated or require dose adjustments in DN patients to prevent hypoglycemia (Rangaswami et al., 2020). A number of glucose-lowering medications are available, but only a fraction of them can be used safely in CKD (Xie et al., 2020). There remains an unmet need for innovative treatment strategies to prevent, arrest, treat, and reverse DN.

TCM has a long history of treating diseases in China and other Asian countries, including Japan and Korea, and several herbal medications have been reported as being effective in treating DN (Tang et al., 2021). Analyzing the evolution of the TCM syndrome of DN, they were all deficient in root and excelled in branch. DN with yin and yang deficiency is an important subject of pathogenesis throughout the course of disease (Lu et al., 2004). Kidney-yin deficiency results in the dysregulation of lipid and glucose metabolism (Chen et al., 2021). ZGW, a classic traditional medicine to treat kidney-yin deficiency, has been described in *Jingyue Quanshu* by Jingyue Zhang (1563–1640 A.D.). ZGW is also indicated in the Chinese Pharmacopoeia for treating kidney-yin deficiency and associated with glycemic control in gestational diabetes (Xu et al., 2019). Meanwhile, the medicine alleviated maternal kidney-yin deficiency-induced thymic epithelial cell dysfunction (Chen et al., 2021). Protecting podocytes against hyperglycemia can prevent diabetes-associated albuminuria without the need of restoring normal levels of glucose. In the present study, ZGW did not improve the blood glucose in db/db mice but alleviated kidney function, suggesting its vital role in podocytes.

A large body of evidence describes the relationship between podocytes and albuminuria (Feng et al., 2021; Makino et al., 2021). Since podocytes are unable to divide, injury and dysfunction of these cells lead to the leakage of protein into urine. The mechanisms underlying podocyte apoptosis are the focus of intense research. High doses of albumin contribute to podocyte loss and apoptosis (Cao et al., 2016). Here, changes in renal function, renal histopathology, podocyte loss and apoptosis were observed in db/db mice and high glucose-stimulated podocytes. ZGW treatment ameliorated these changes and increased the expression of the podocyte marker WT-1 in diabetic mice.

High glucose treatment results in cytoskeleton rearrangements through mitochondrial dysfunction (Wang et al., 2022), and high glucose induces apoptosis by stimulating ROS production (Fan et al., 2019). Complex I and III, as two major sources of cellular ROS, generate ROS when electron transport is slowed by high mitochondrial membrane potential. Proper levels of ROS play a vital role in signaling pathways, while excess ROS production overwhelms the cellular antioxidant capacity and induces cell apoptosis (Begum et al., 2022). The present study showed high glucose induced ROS generation in podocytes, which was in accordance with previous studies (He et al., 2022). ZGW dose-dependently reduced ROS production and prevented podocyte apoptosis. Examination of the effects of ZGW on the activities of antioxidant enzymes (CAT and SOD) showed the decreases of both CAT and SOD activities in podocytes exposed to high glucose, which was effectively inhibited by ZGW treatment and further confirmed *in vivo* study. Thus, ZGW inhibited podocyte apoptosis induced by high glucose and improved renal injury in db/db mice *via* an antioxidant signaling pathway.

The activation of caspase enzymes is an important biochemical change in apoptosis. Activation of caspase-3, which operates as the key effector enzyme in cell apoptosis through death-receptor and mitochondria, is the vital determinant of apoptosis (Van Opdenbosch and Lamkanfi, 2019). The experimental results revealed the activation of cleaved caspase-3 was a result of high glucose, indicating the caspase pathway was involved in the process of high glucose-induced apoptosis in podocytes. ZGW reduced caspase-3 activation *in vitro* and *in vivo*.

MAPK, as an important intracellular transduction signal in renal cell death and the development of DN, has three major subfamilies, namely, ERK1/2, JNK, and p38 MAPK, which are shown to be related to the pathogenesis of DN (Nicholas et al., 2010; Loeffler et al., 2011). In our study, high glucose, instead of upregulating phospho-JNK and total JNK, significantly enhanced the phosphorylation levels of p38 in podocytes and diabetic mice and was effectively suppressed by ZGW. Therefore, ZGW is likely to inhibit podocyte apoptosis by regulation of the p38 MAPK pathway.

In summary, the present study suggests that ZGW may be a potential therapeutic approach for DN. ZGW inhibited apoptosis *in vivo* and *in vitro* through reducing ROS generation, apoptosis and regulating phospho-p38 signaling. Therefore, a potential protective mechanism of ZGW toward the kidney was confirmed, which strengthens the therapeutic rationale for treating DN.

## Data Availability

The original contributions presented in the study are included in the article/Supplementary Material; further inquiries can be directed to the corresponding authors.
