# Supplementary material for: Zuogui Wan ameliorates high glucose-induced podocyte apoptosis and improves diabetic nephropathy in db/db mice
Source: Front Pharmacol. 2022 Nov 1;13:991976. doi: 10.3389/fphar.2022.991976 (PMC9663993; doi:10.3389/fphar.2022.991976)
Supplement: Supplementary file 1 [file DataSheet2.PDF]

Dot plot analysis shows the region of podocytes (R1), based on their forward scatter (FSC) and side scatter (SSC) characteristic. Dot plot analysis of podocytes (R1 region) shows intact cells (Annexin V-negative, PI-negative), early apoptotic cells (Annexin V-positive, PI-negative), apoptotic cells (Annexin V-positive and PI-positive) and necrotic cells (Annexin V-negative, PI-positive) in different stimulations on podocytes.

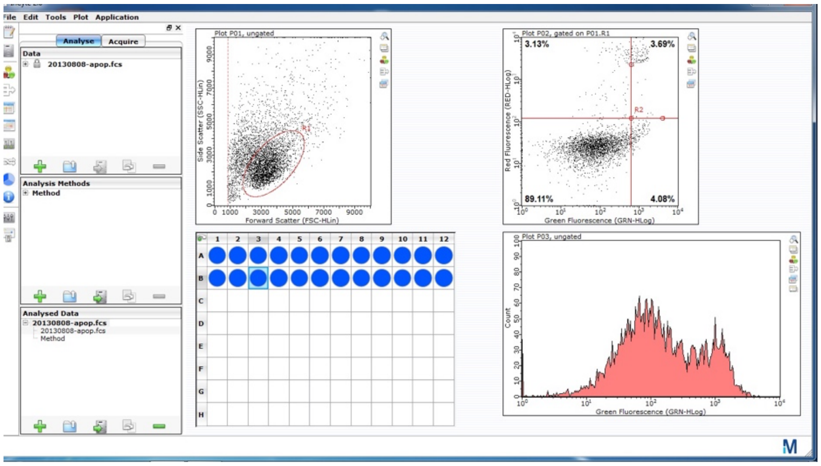

Control-1

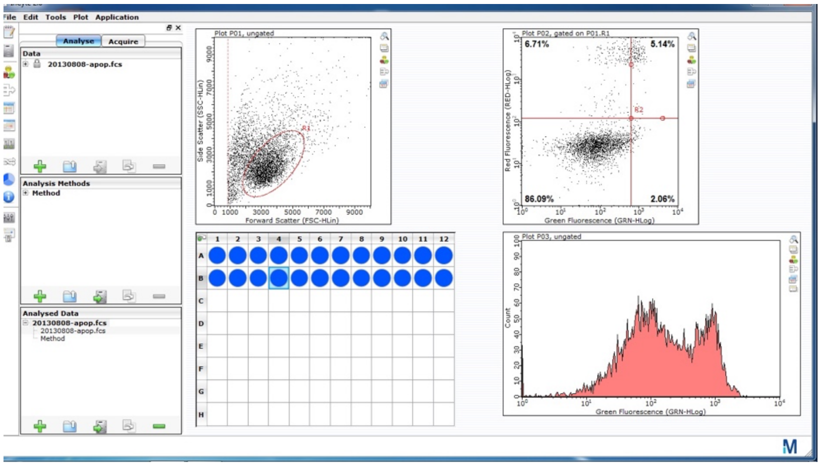

Control-2

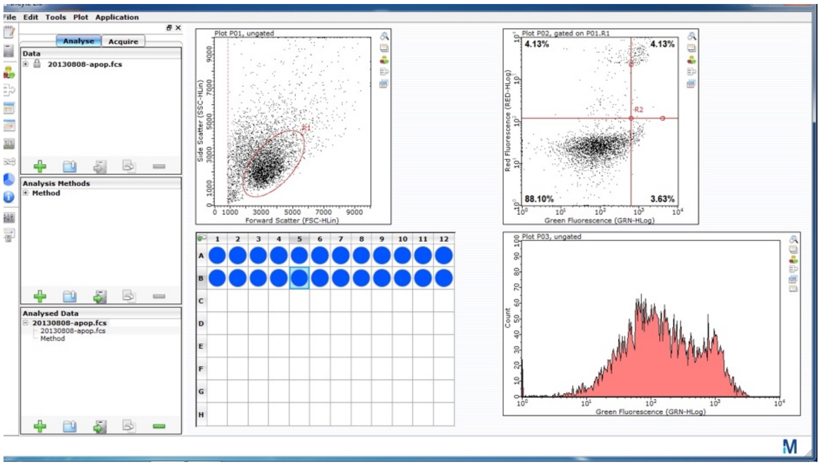

Control-3

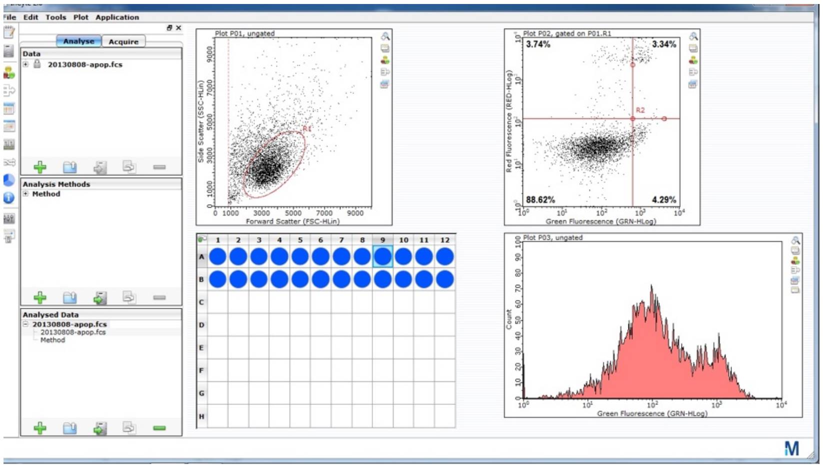

Mannitol-1

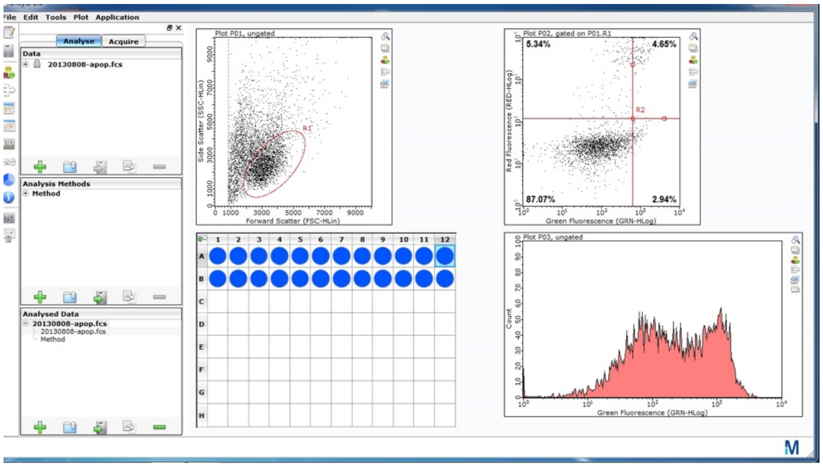

Mannitol-2

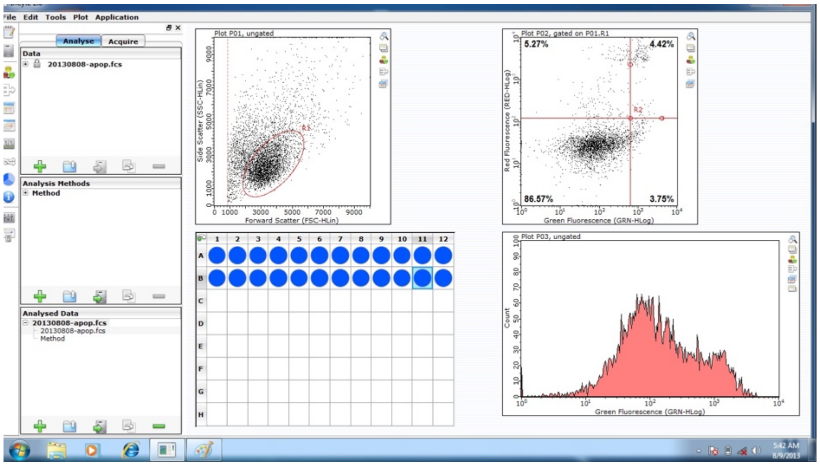

Mannitol-3

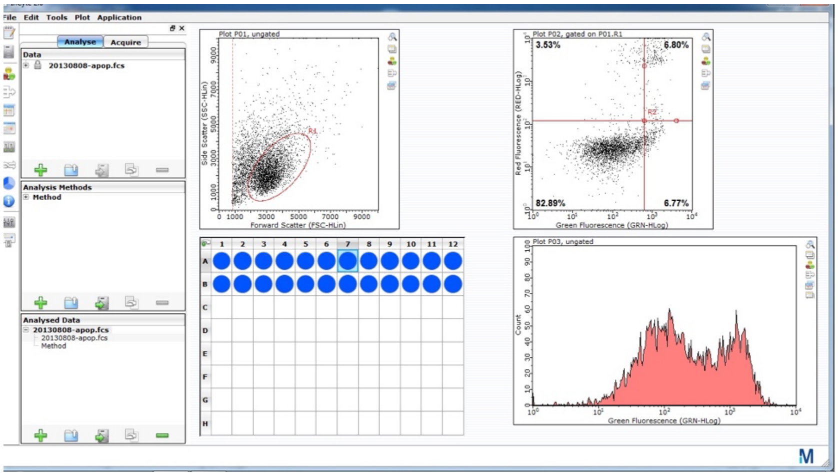

HG-1

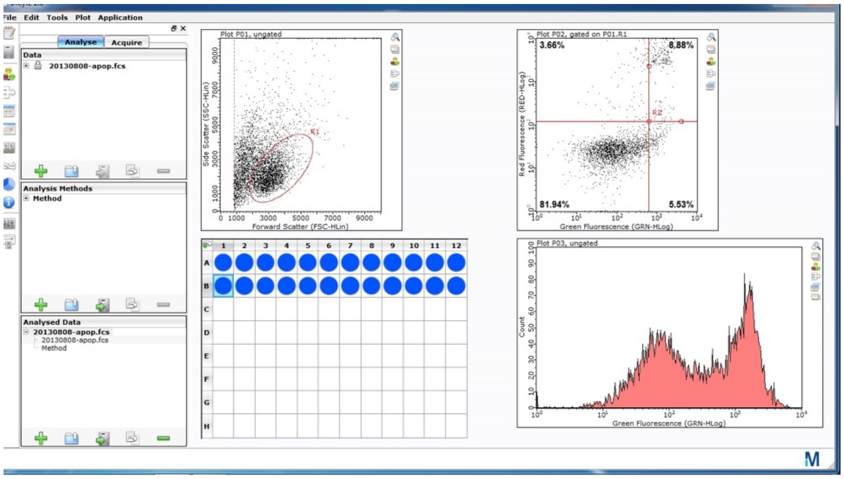

HG-2

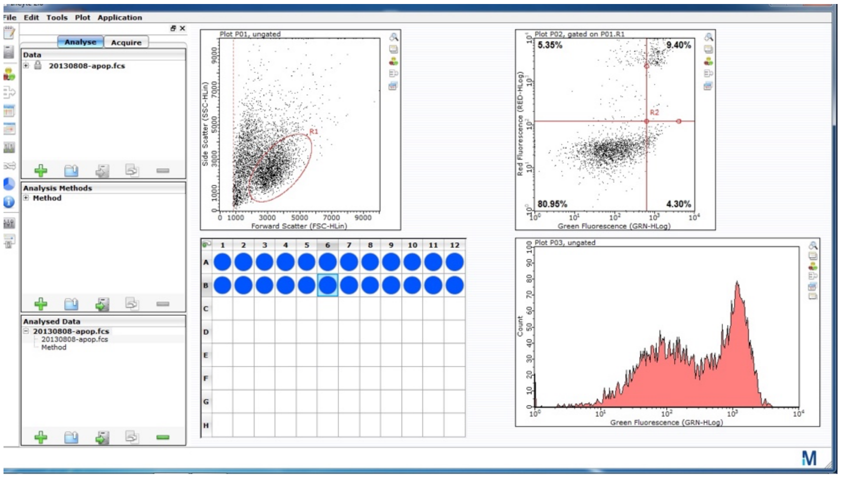

HG-3

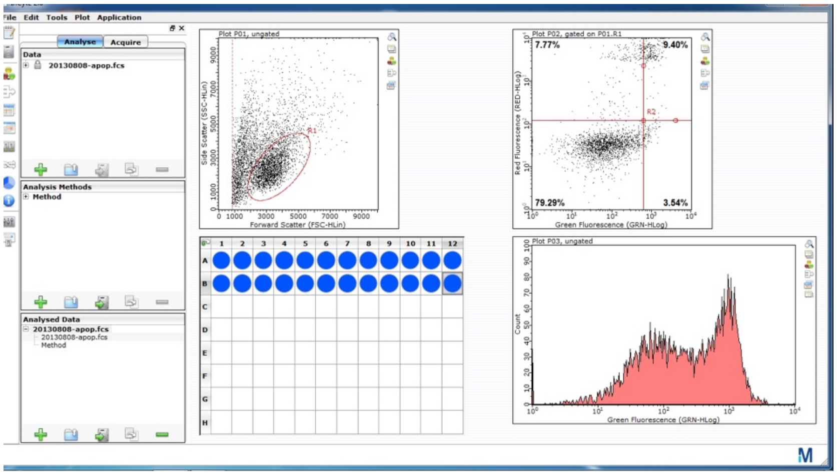

ZWG-100-1

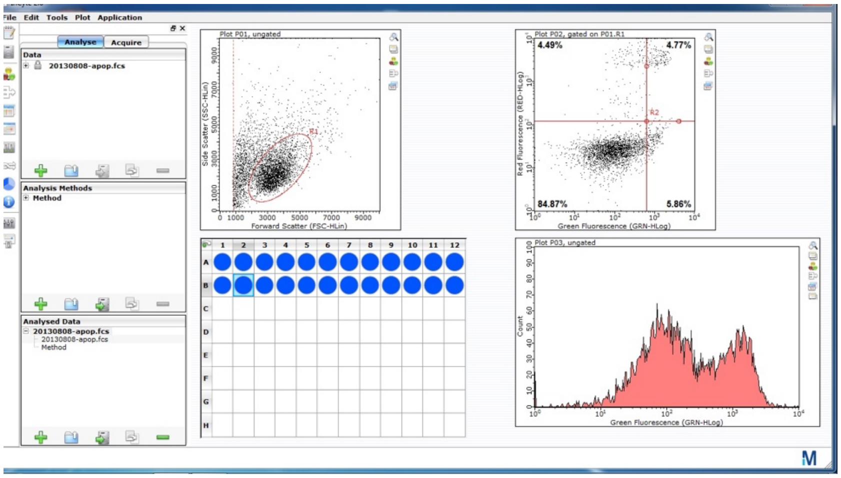

ZWG-100-2

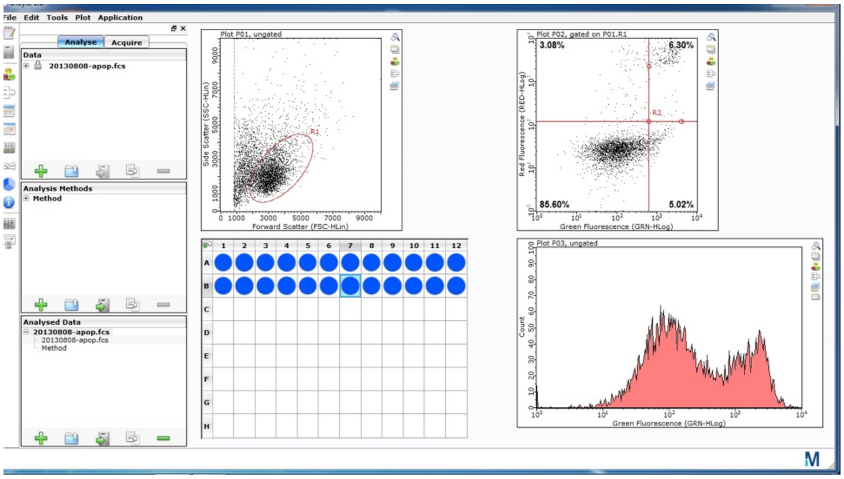

ZWG-100-3

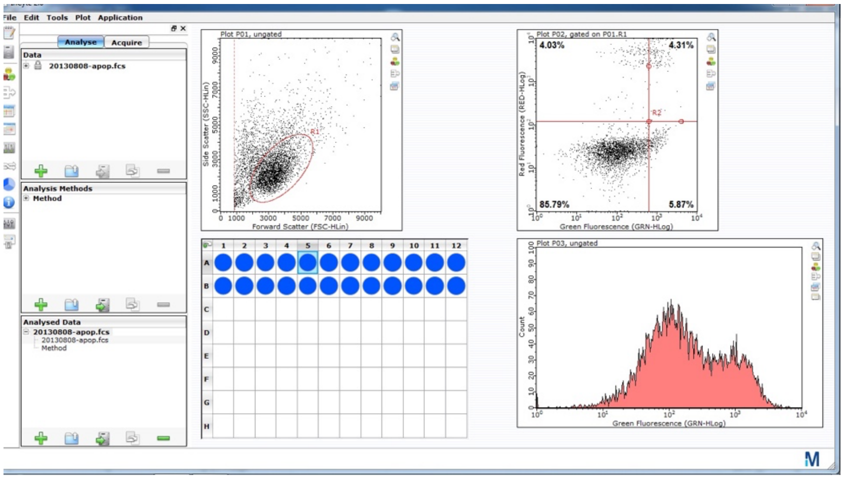

ZWG-300-1

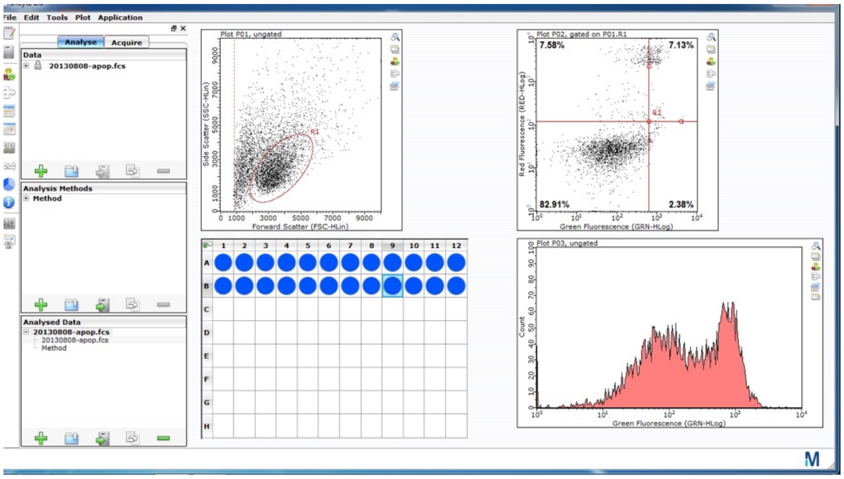

ZWG-300-2

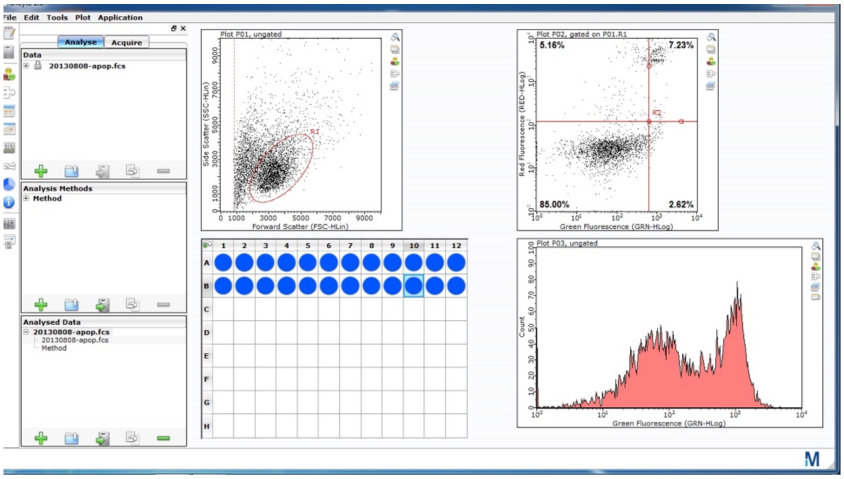

ZWG-300-3

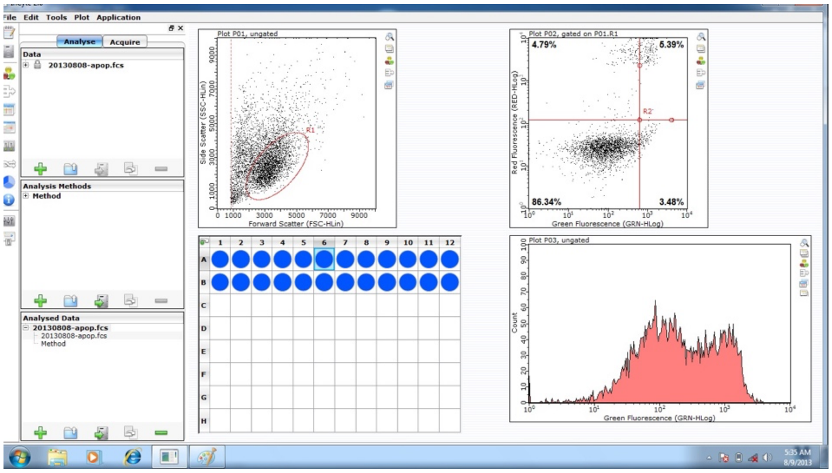

ZWG-1000-1

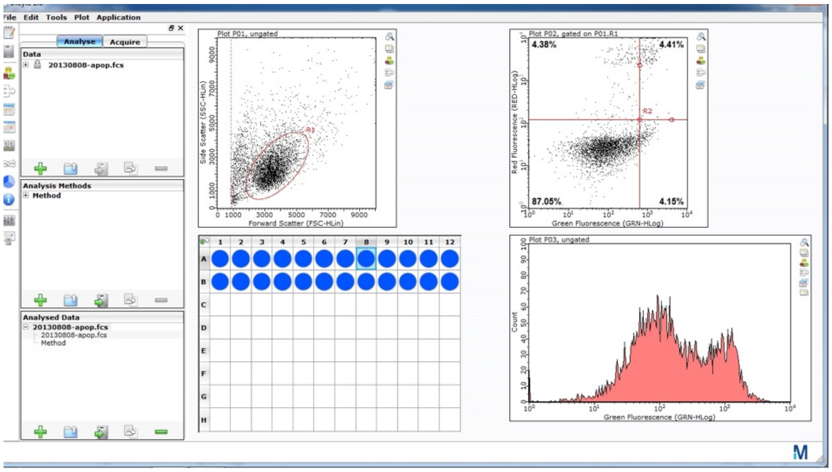

ZWG-1000-2

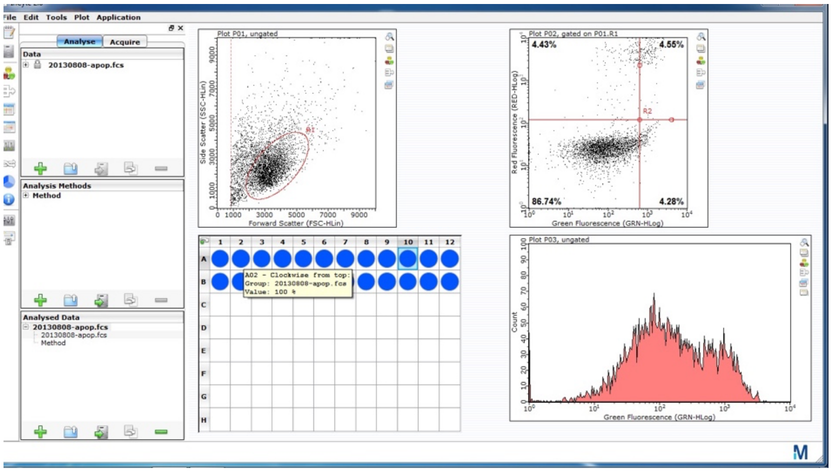
File Edit Tools Plot Application
Analyse
Acquire
Data
20130808-apop.fcs
Analysis Methods
Method
Analysed Data
20130808-apop.fcs
20130808-apop.fcs
Method
Plot P01, ungated
Side Scatter (SSC-HLin)
Forward Scatter (FSC-HLin)
R1
Plot P02, gated on P01.R1
4.43%
4.55%
86.74%
4.28%
R2
Red Fluorescence (RED-HLog)
Green Fluorescence (GRN-HLog)
A02 - Clockwise from top
Group: 20130808-apop.fcs
Value: 100 %
Plot P03, ungated
Count
Green Fluorescence (GRN-HLog)

ZWG-1000-3
