# Supplementary figures and images for: Zuogui Wan ameliorates high glucose-induced podocyte apoptosis and improves diabetic nephropathy in db/db mice
Source: Front Pharmacol. 2022 Nov 1;13:991976. doi: 10.3389/fphar.2022.991976 (PMC9663993; doi:10.3389/fphar.2022.991976)

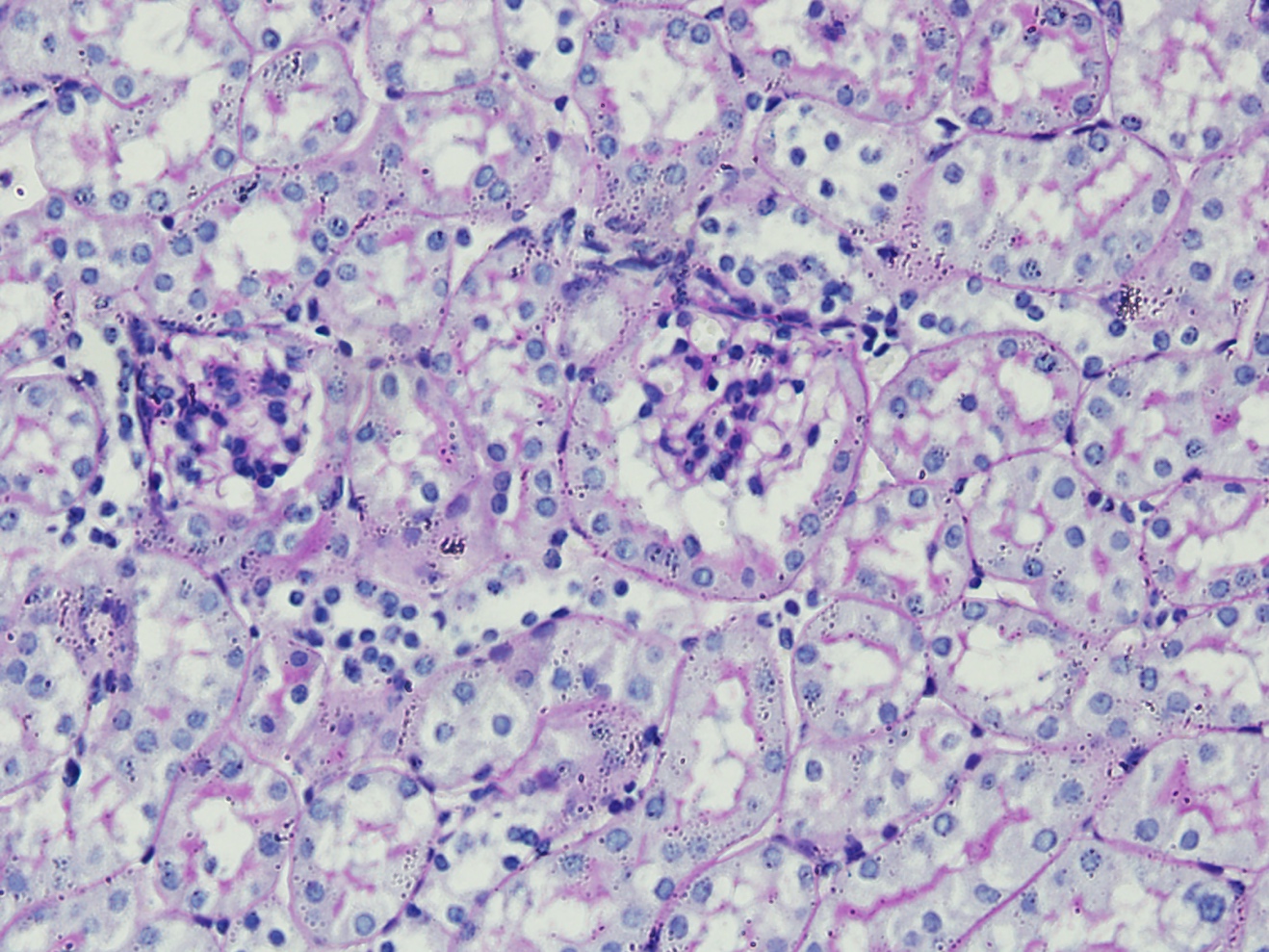


db/m


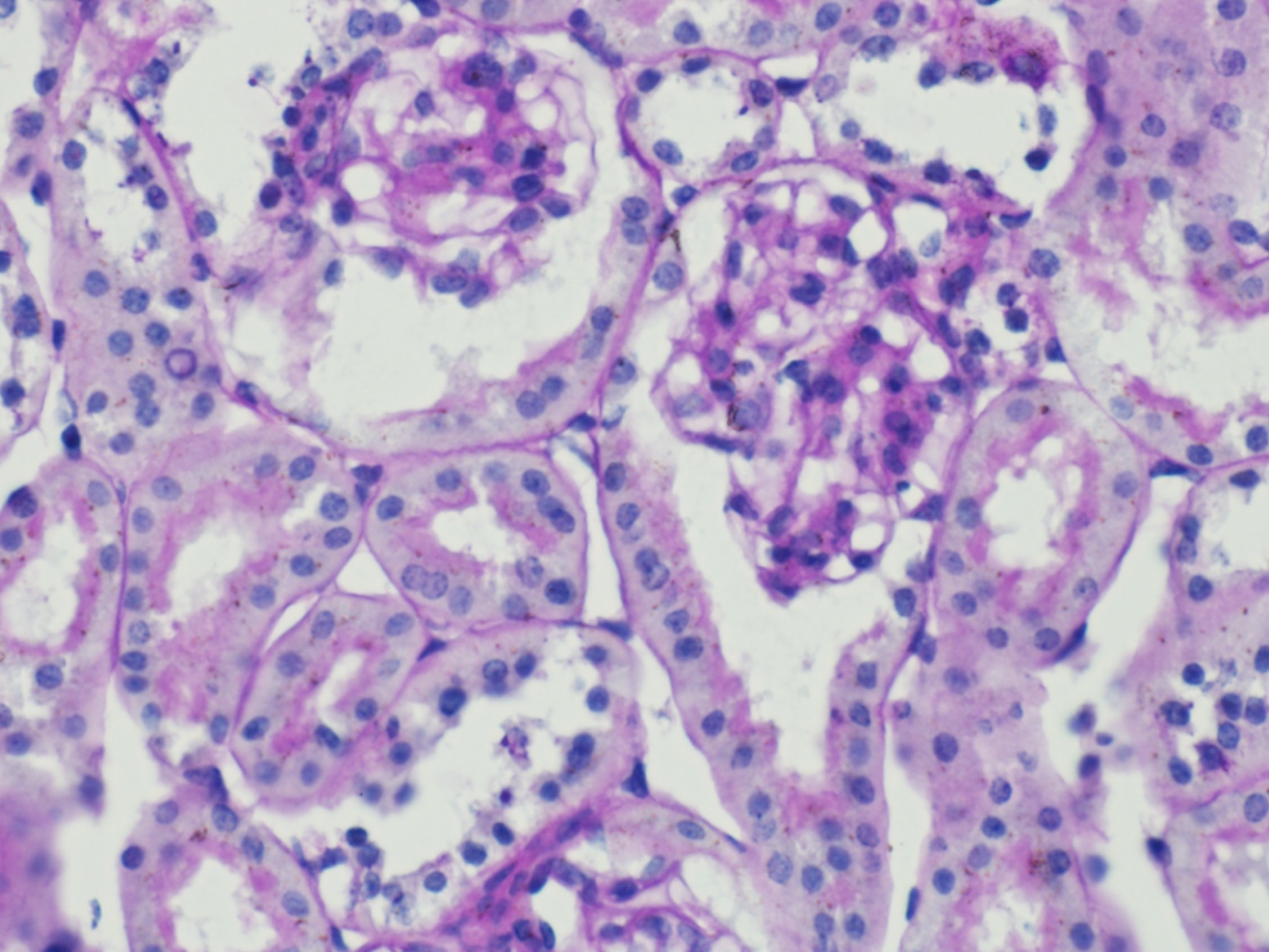


db/db


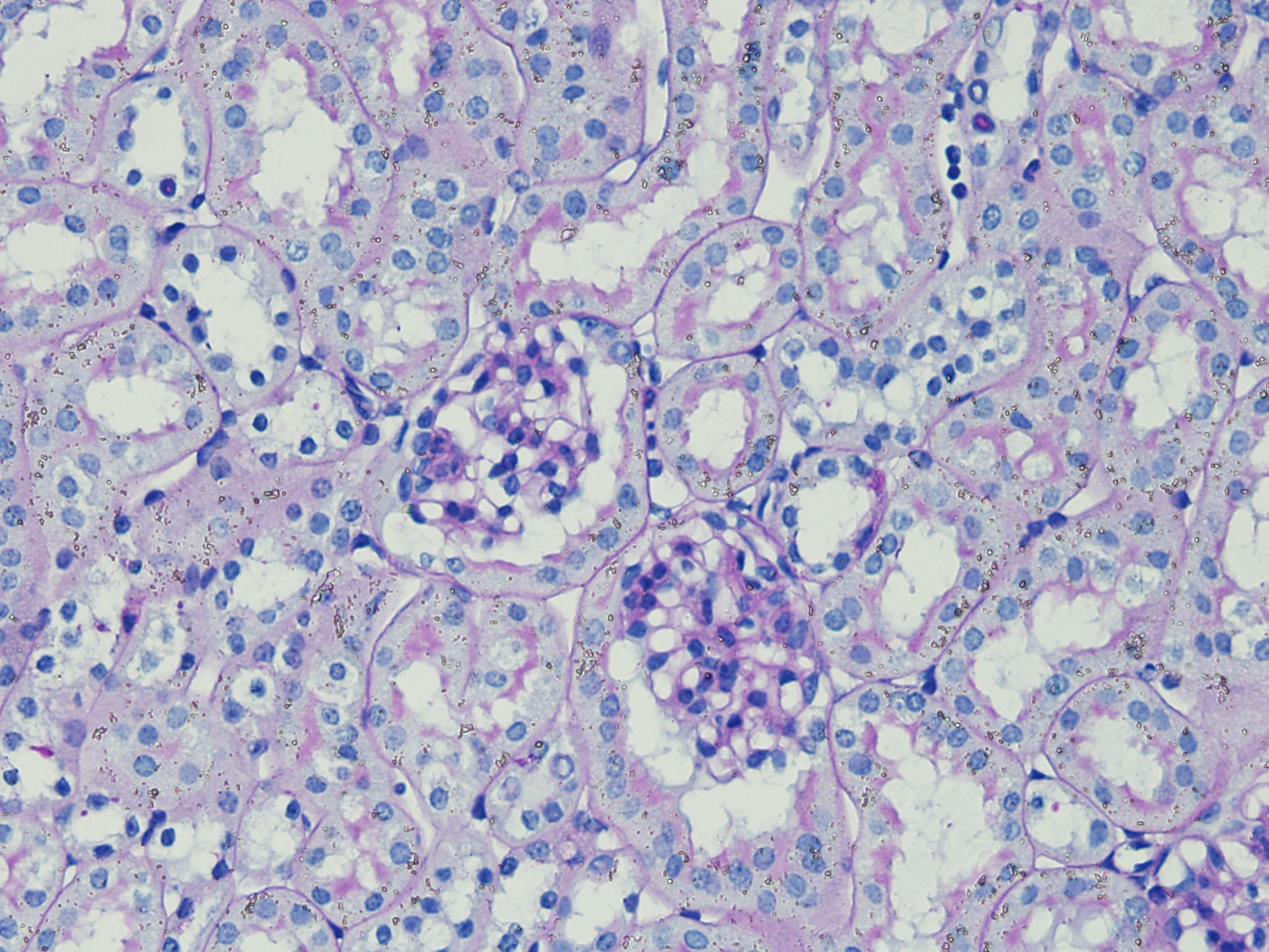


db/db+ZGW

Supplement: Supplementary file 2 [file DataSheet4.DOCX]

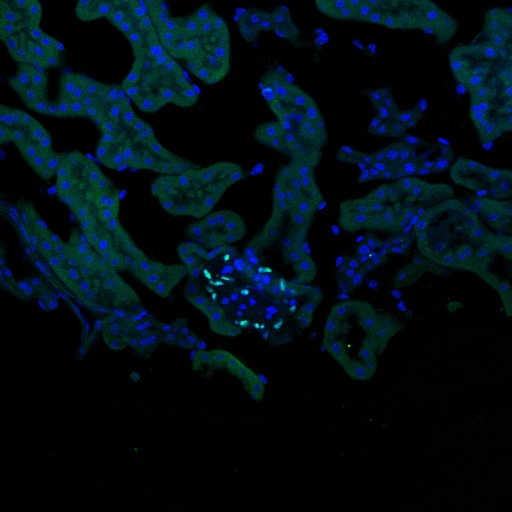


db/m


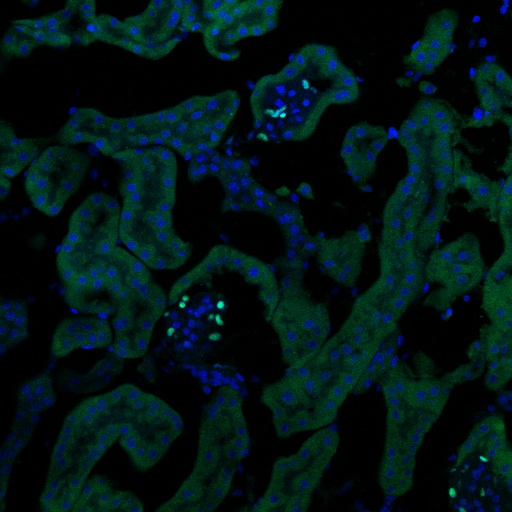


db/db


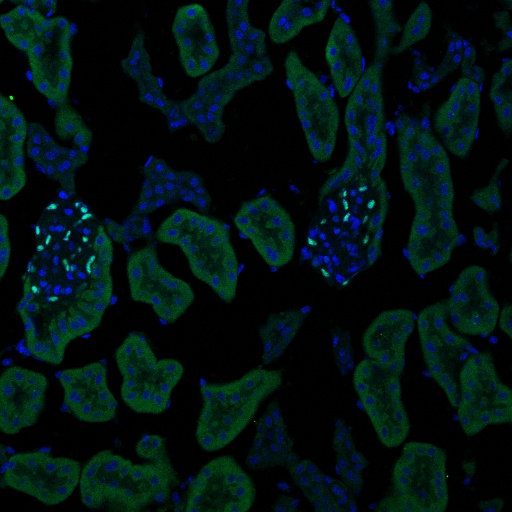


db/db+ZGW

Supplement: Supplementary file 3 [file DataSheet5.DOCX]
