# Supplementary material for: Zuogui Wan ameliorates high glucose-induced podocyte apoptosis and improves diabetic nephropathy in db/db mice
Source: Front Pharmacol. 2022 Nov 1;13:991976. doi: 10.3389/fphar.2022.991976 (PMC9663993; doi:10.3389/fphar.2022.991976)
Supplement: Supplementary file 5 [file DataSheet1.PDF]

Two different protein markers were used for the whole experiments. They were listed as follows: 161-0374 (Bio-Rad) and 26616 (Thermo Scientific)

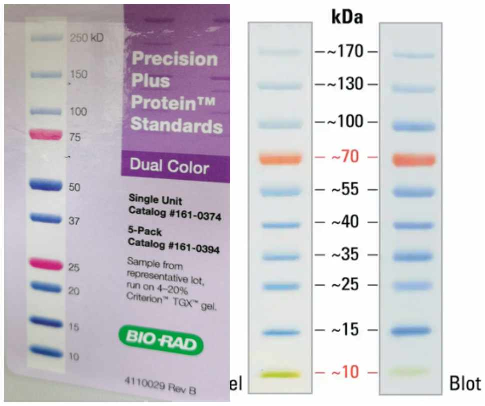

In vivo study

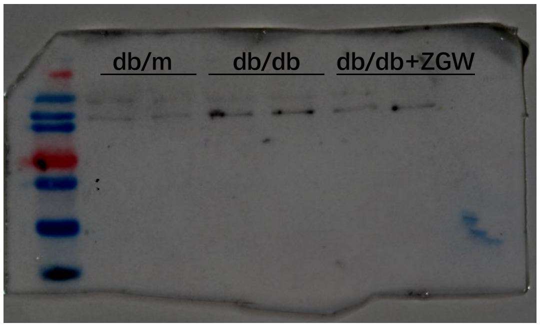

phospho-p38 (43 KDa)

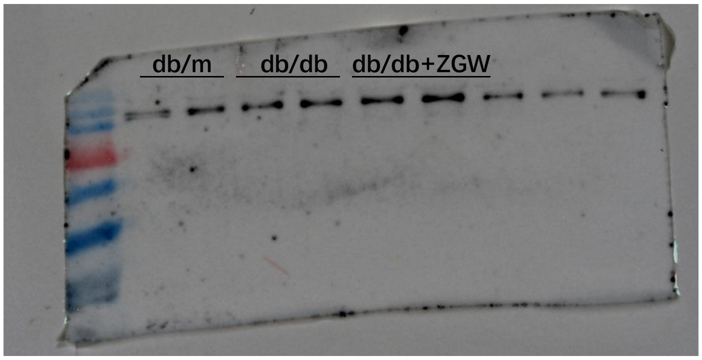

p38 (43 KDa)

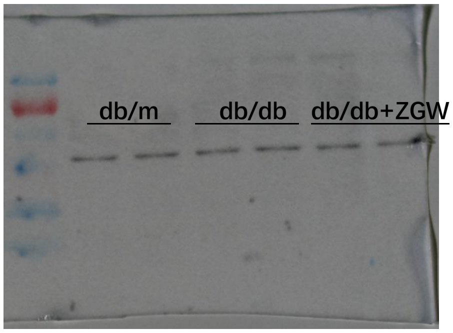

phospho-JNK (46/54 KDa)

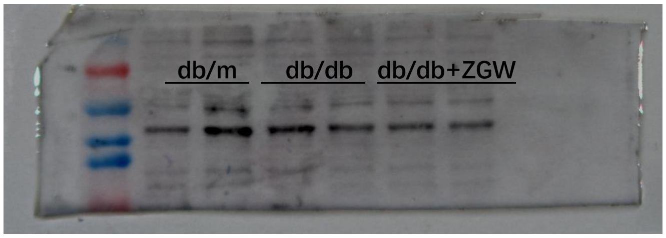

JNK(46/54 KDa)

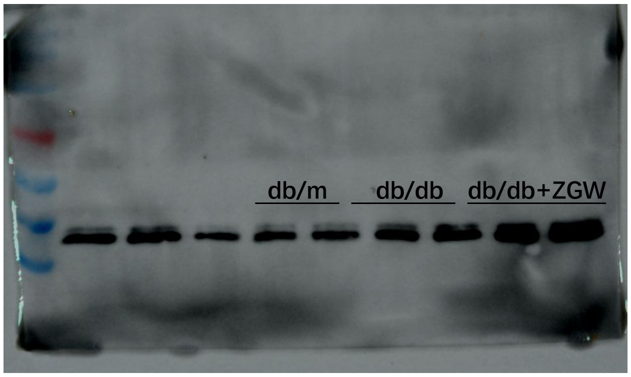

phospho-ERK (42/44 kDa)

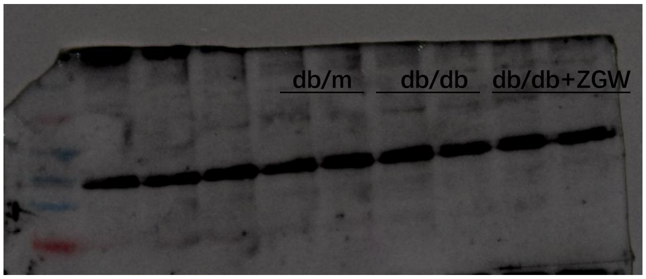

ERK (42/44 kDa)

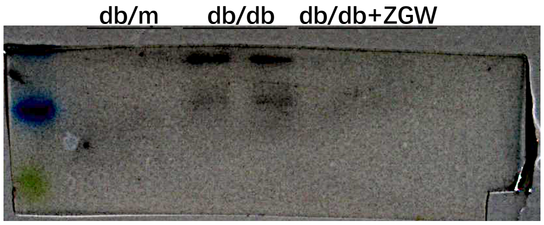

Caspase-3 (17 Kda)

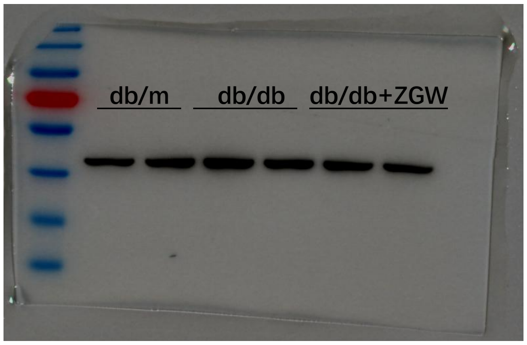

ß-actin (42kDa)

In vitro study

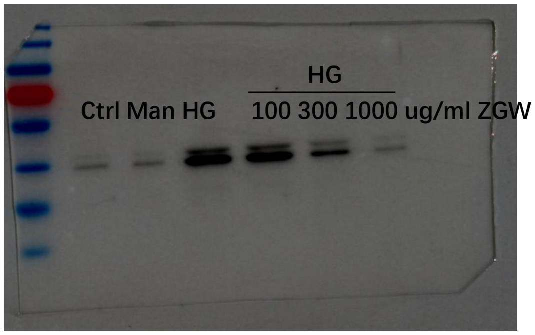

phospho-p38 (43 KDa)

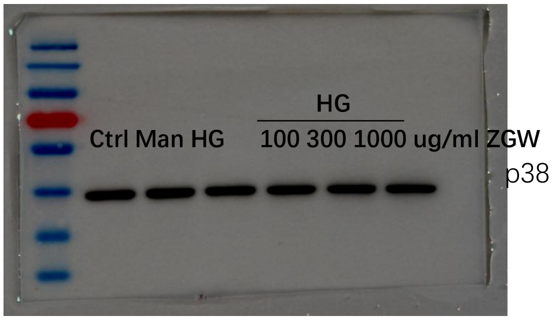

p38 (43 kDa)

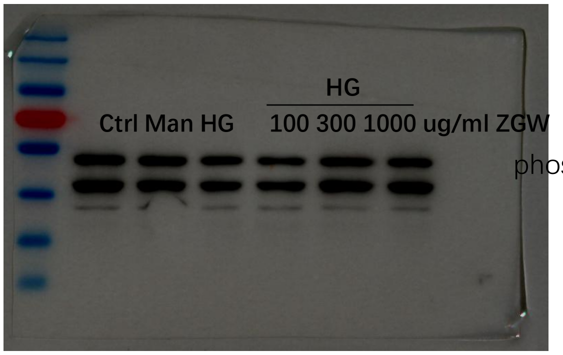

phospho-JNK (46/54 kDa)

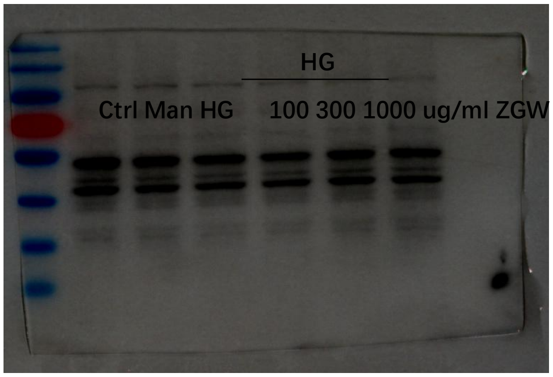

JNK (46/54 kDa)

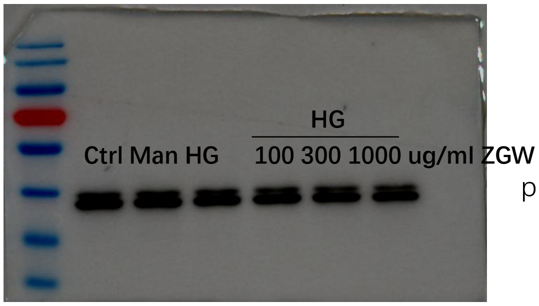

phospho-ERK (42/44 kDa)

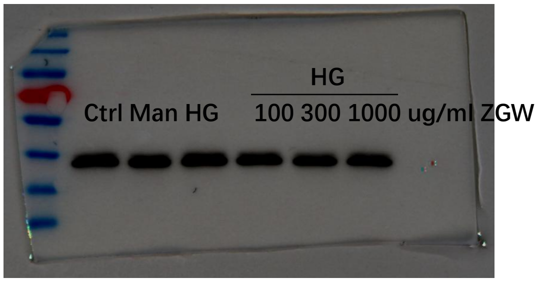

ERK (42/44 kDa)

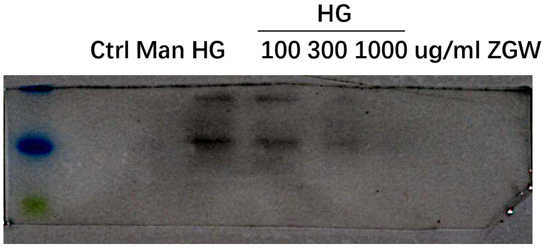

Caspase-3 (17kDa)

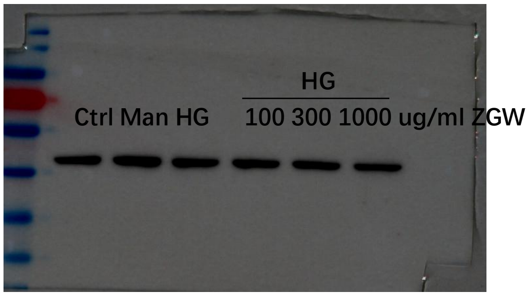

ß-actin (42kDa)
